# Targeting ARPC1B ^+^ Cancer Stem Cells to Sensitise Pancreatic Cancer to Gemcitabine Treatment

**DOI:** 10.1111/cpr.70125

**Published:** 2025-09-03

**Authors:** Yang Wu, Jianpeng Zhang, Weixiong Zhu, Xinrui Zhu, Yi Liu, Xin Wang, Tianyu Zhao, Chun Zhang, Zili Zhang, Wenjie Shi, Run Shi, Zhaokai Zhou, Shaohui Xu

**Affiliations:** ^1^ Pancreas Center The First Affiliated Hospital of Nanjing Medical University Nanjing China; ^2^ Department of Urology The First Affiliated Hospital of Guangzhou Medical University Guangzhou China; ^3^ The Second Clinical Medical College Lanzhou University Lanzhou China; ^4^ Department of Hepatobiliary and Pancreatic Surgery The Third Affiliated Hospital of Soochow University Changzhou Jiangsu China; ^5^ Department of Oncology The Second Hospital of Dalian Medical University Dalian China; ^6^ Department of Oncology The Affiliated Cancer Hospital of Nanjing Medical University, Jiangsu Cancer Hospital, Jiangsu Institute of Cancer Research Nanjing China; ^7^ Institute of Social Medicine and Epidemiology Medical University of Graz Graz Austria; ^8^ Department of Gastroenterology Affiliated Hospital of Nanjing University of Chinese Medicine Nanjing China; ^9^ State Key Laboratory on Technologies for Chinese Medicine Pharmaceutical Process Control and Intelligent Manufacture Nanjing University of Chinese Medicine Nanjing China; ^10^ Molecular and Experimental Surgery, Clinic for General‐, Visceral‐, Vascular‐ and Transplantation Surgery Medical Faculty and University Hospital Magdeburg, Otto‐von‐Guericke University Magdeburg Germany; ^11^ Department of Oncology The First Affiliated Hospital of Nanjing Medical University Nanjing China; ^12^ Department of Urology The Second Xiangya Hospital of Central South University Changsha China; ^13^ Institute of Functional Nano & Soft Materials (FUNSOM) Soochow University Suzhou China

## Abstract

ARPC1B^+^ cancer stem cells (CSCs) in pancreatic cancer are identified as a subpopulation resistant to gemcitabine. In our study, drug repositioning, molecular docking, and surface plasmon resonance (SPR) technique jointly revealed that CK‐636 can directly target ARPC1B protein with high affinity. In vitro cytotoxicity, ex vivo organoid cultures, in vivo xenograft and orthotopic gemcitabine‐resistant pancreatic cancer model demonstrated that combination therapy of gemcitabine plus CK‐636 showed a superior anti‐tumor effect compared with gemcitabine monotherapy. Our study demonstrated that CK‐636 can act as a rational adjuvant to overcome gemcitabine resistance in pancreatic cancer therapy.

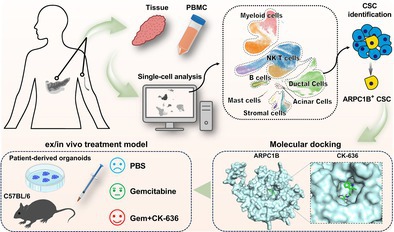

To the Editor,

Pancreatic cancer is one of the most aggressive and lethal malignancies, and it poses a significant challenge globally with a 5‐year overall survival rate of less than 10% [1]. The high mortality rate of pancreatic cancer results from late diagnosis, rapid progression, and resistance to conventional therapies. Currently, surgical resection remains the only potentially curative option for pancreatic cancer; however, fewer than 20% of patients are eligible due to advanced disease stages [1]. For advanced or metastatic cases, gemcitabine‐based chemotherapy has long been a primary treatment choice, but its efficacy is severely limited by the intrinsic and acquired chemoresistance of pancreatic cancer cells [2]. Given these challenges, researchers have increasingly focused on identifying the underlying mechanisms of chemoresistance to develop more effective and personalised therapeutic strategies.

Cancer stem cells (CSCs) become attractive to researchers due to their critical role in cancer therapy resistance [3]. These cells possess self‐renewal capacity and multipotency, which enable them to drive tumour initiation, facilitate malignant progression, and acquire resistance to chemotherapy [4]. In pancreatic cancer, CSCs are characterised by some specific surface markers such as CD133 and CD44, distinguishing them from non‐stem cancer cells [5]. Emerging evidence suggests that not all CSCs are functionally equivalent, and the heterogeneity of the CSC populations may exhibit distinct biological functions and account for differential responses to treatment [6]. Therefore, we aim to investigate the specific subpopulation of pancreatic CSCs that are particularly refractory to gemcitabine, and further evaluate their potential as a novel biomarker and therapeutic target for gemcitabine‐resistant pancreatic cancer.

## 1 ARPC1B ^+^ CSC Population was Identified Resistant to Gemcitabine

**FIGURE 1:**
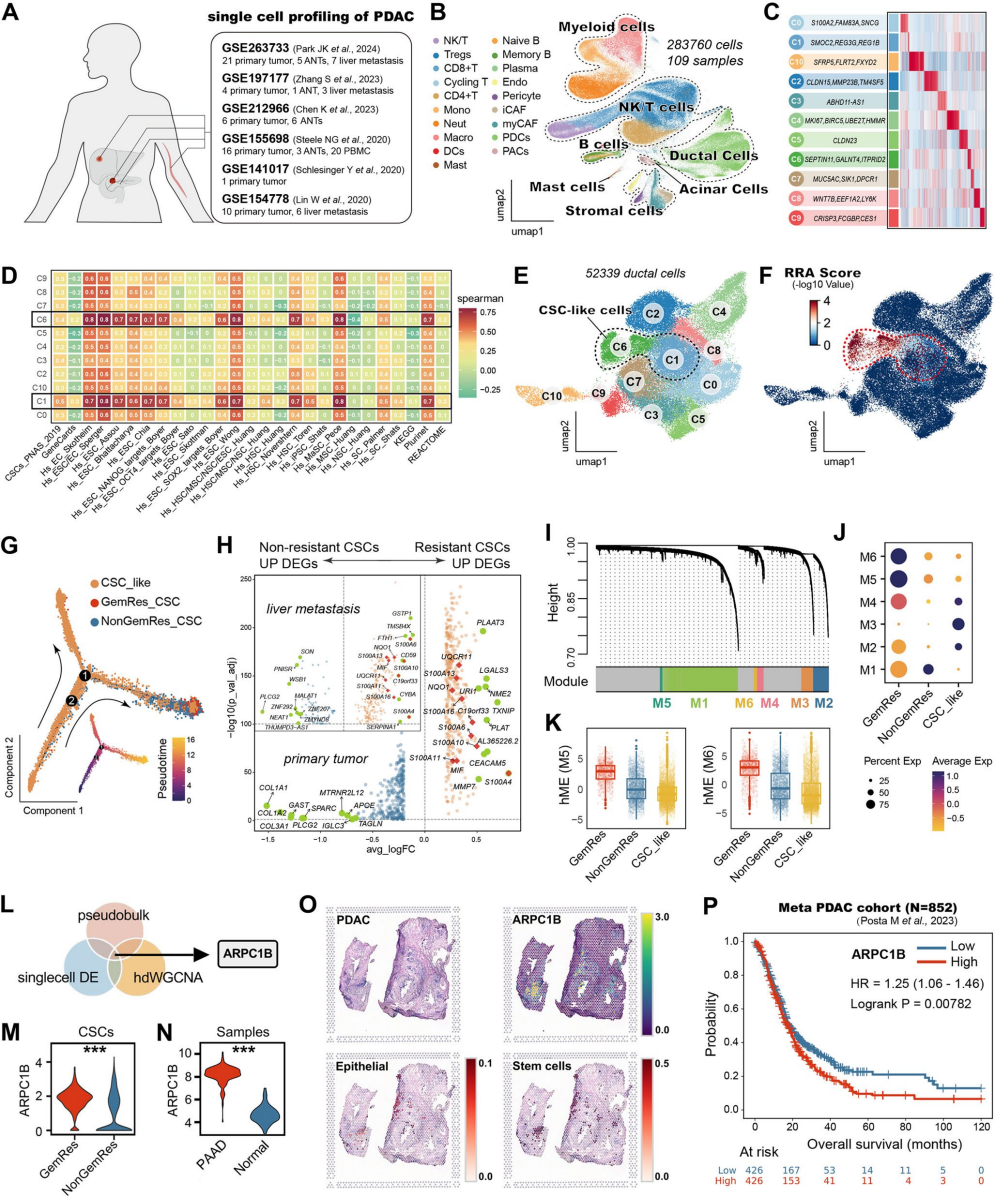
ARPC1B^+^ CSC population was identified as resistant to gemcitabine. (A) Six scRNA‐seq datasets of pancreatic ductal adenocarcinoma (PDAC) were collected and integrated for further investigation. (B) Major cell types were identified in the PDAC tumour microenvironment. (C) Specific marker genes for each subcluster of pancreatic ductal cells (PDCs). (D) Stemness scores of different PDC clusters were assessed by stemness signatures from 27 published studies, and clusters C6 and C1 exhibited the highest stemness. (E) C1 and C2 are considered CSC‐like cell clusters. (F) Both C1 and C6 exhibited a high correlation with gemcitabine resistance scores. (G) Pseudotime analysis of tumour cell evolution illustrates the evolutionary trajectory from non‐gemcitabine‐resistant to resistant phenotypes, with CSC‐like cells serving as the origin. (H) Differentially expressed genes in gemcitabine‐resistant CSCs and non‐resistant cells. (I) Six distinct gene co‐expression modules were identified using the hdWGCNA algorithm. (J, K) M5 and M6 displayed significant activation in the gemcitabine‐resistant group. (L) Identification of ARPC1B as an overlapping hub gene from three approaches: Pseudobulk, single‐cell DE, and hdWGCNA. (M) ARPC1B is significantly elevated in gemcitabine‐resistant CSCs. (N) ARPC1B is significantly elevated in pancreatic cancer samples compared to normal tissues. (O) The spatial transcriptomics (ST) analysis of a PDAC sample demonstrated that ARPC1B is highly enriched in malignant epithelia, particularly in stem cells. (P) Higher expression of ARPC1B indicates worse overall survival in a meta PDAC cohort. ***p < 0.001.

An increasing number of studies have integrated single‐cell RNA‐sequencing (scRNA‐seq) and bulk tissue RNA‐seq to identify potential targets or cell subpopulations in order to facilitate a deeper understanding of cancer biology, disease progression, and treatment responses [7]. In this study, we initially collected 6 scRNA‐seq datasets of pancreatic ductal adenocarcinoma (PDAC). In summary, these datasets obtain 15 pieces of adjacent normal tissues (ANTs), 58 pieces of primary tumours, 16 pieces of liver metastasis, and 20 pieces of peripheral blood mononuclear cells (PBMCs) samples (Figure 1A). Details of scRNA‐seq data integration and data processing are provided in the supplementary file. After quality control and batch correction, a total of 283,760 high‐quality cells and 19,658 genes were retained for further analysis. A total of 7 main cell types and 19 subtypes were identified, including immune cells such as natural killer T cells (NK/T), regulatory T cells (Tregs), CD8^+^ T cells, cycling T cells, CD4^+^ T cells, monocytes, neutrophils, macrophages, dendritic cells (DCs), and mast cells; epithelial cells such as pancreatic ductal cells (PDCs) and acinar cells (PACs); and stromal cells including pericytes, inflammatory cancer‐associated fibroblasts (iCAFs), and myofibroblastic cancer‐associated fibroblasts (myCAFs) (Figure 1B). The expression landscape of gene markers for these cell types is shown in Figure S1. PDCs exhibited intra‐tumoral heterogeneity and were further divided into 11 clusters (C0‐C10) with marker genes (Figure 1C). To identify high‐confidence CSCs, epithelial cell subtypes were independently assessed using stemness signatures curated from 27 published studies. The gene sets are described in Table S1. Through the Robust Rank Aggregation algorithm (RRA), cells with consensus scores below 0.05 exhibited significant stemness enrichment across signatures. Among the clusters, C6 displayed the highest stemness, followed by C1, while other clusters demonstrated negligible stemness activity (Figure 1D). Therefore, C1 and C6 were considered as CSC‐like clusters (Figure 1E). Meanwhile, correlation analyses between the clusters and upregulated gene sets associated with gemcitabine resistance revealed that both C1 and C6 exhibited a higher correlation with gemcitabine resistance scores (Figure 1F). Furthermore, pseudotime analysis indicated that CSC‐like cells serve as the origin of tumour cells, illustrating an evolutionary trajectory in which initially non‐gemcitabine‐resistant cells gradually evolve into a resistant phenotype (Figure 1G). These results demonstrated that cancer stemness plays a critical role in the development of drug resistance, and interventions aimed at targeting the drug‐resistant CSCs could potentially enhance the efficacy of chemotherapy and overcome drug resistance.

Subsequently, we combined differential gene expression analysis and the hdWGCNA algorithm to identify the gemcitabine‐resistant CSC subpopulation. In the differential gene expression analysis, compared to non‐resistant groups in primary PDAC and hepatic metastatic tissues, several genes were significantly elevated in the primary and metastatic lesions, including some S100 calcium‐binding proteins (S100A4, S100A6, S100A10, S100A11, S100A13, S100A16), MIF, NQO1, URI1, and C19orf33 (Figure 1H). The hdWGCNA algorithm was employed with a soft‐thresholding power (β = 8) at a scale‐free topology fit index of 0.90 (Figure S2), and six distinct gene co‐expression modules were identified (Figure 1I). Subsequent evaluation of module eigengene scores across cellular clusters revealed that modules M5 and M6 displayed significant activation in the gemcitabine‐resistant group (Figure 1J,K). These findings indicated the genes involved in modules M5 and M6 as hub candidates that are correlated with gemcitabine resistance. Subsequently, three approaches (pseudobulk, singlecell DE, and hdWGCNA) were integrated (hub genes are summarised in Table S2) and identified ARPC1B as an overlapping hub gene (Figure 1L), showing significant upregulation in gemcitabine‐resistant CSCs compared to non‐resistant CSCs and in tumour samples versus normal tissues (Figure 1M,N). The spatial transcriptomics (ST) analysis of a PDAC sample (sample id: VISDS000994, available in the CROST database, https://ngdc.cncb.ac.cn/crost/home) [8] validated that ARPC1B is highly enriched in malignant epithelia, particularly in stem cells (Figure 1O). Furthermore, a total of 852 PDAC patients from a multicenter‐derived meta‐cohort (Kaplan–Meier Plotter platform, https://kmplot.com/analysis/) [9] were included and analysed, and we observed that patients with higher ARPC1B expression exhibited significantly shorter overall survival (HR = 1.25, *p* < 0.01), which further indicated ARPC1B as a potential biomarker of poor prognosis and a promising therapeutic target for pancreatic cancer (Figure 1P). In addition, we employed a deconvolution approach to assess the resistance score for each patient. We evaluated stemness scores by integrating deconvolution results from 27 stemness‐related gene sets. Subsequently, we analysed the overall survival of pancreatic cancer patients based on multiple dimensions, including the degree of gemcitabine resistance, tumour stemness, and ARPC1B expression levels. We observed that high ARPC1B expression and elevated stemness are significantly associated with worse survival, particularly among patients who are potentially gemcitabine‐resistant (Figure S3).

## 2 ARPC1B Correlates With a Higher Mutation Burden and Increased Intra‐Tumour Heterogeneity in Pancreatic Cancer

Subsequently, we used TCGA‐PAAD transcriptome data to reveal the relationship between ARPC1B and cancer‐related biological functions and other cancer hallmarks. Firstly, we obtained the stemness index (mRNAsi) for each pancreatic cancer sample from the study of Malta et al. [10]. As shown in Figure S4A, a total of 177 PAAD samples were categorised to 4 groups based on their stemness index and ARPC1B expression levels: G1: Stemness^low^/ARPC1B^low^; G2: Stemness^low^/ARPC1B^high^; G3: Stemness^high^/ARPC1B^low^; G4: Stemness^high^/ARPC1B^high^. Using ssGSEA algorithm, the geneset “TOOKER_GEMCITABINE_RESISTANCE_UP” retrieved from the Human Molecular Signatures Database (MSigDB) [11] was used to quantify the gemcitabine resistance for each cancer sample. Furthermore, we observed that the ssGSEA scores of “gemcitabine resistance” were significantly elevated in the Stemness^high^/ARPC1B^high^ group compared to the Stemness^low^/ARPC1B^low^ group (Figure S4B). Using the “aPEAR” R package, we explored the biological processes underlying Stemness^high^/ARPC1B^high^ samples. The biological enrichment network demonstrated that the most significant features were labelled as “cell differentiation”, “ATP synthesis coupled electron transport”, and “mitotic nuclear envelope reassembly” etc. (Figure S4C). Subsequently, we integrated and visualised the expression levels of ARPC1B, stemness scores, somatic mutations, and clinicopathological characteristics as a landscape plot (Figure S4D). The most frequently mutated genes in the cohort were KRAS (65%), TP53 (61%), SMAD4 (21%), CDKN2A (21%), and TNN (15%). We compared somatic mutation frequencies between the G4 and G1 groups and found that the G4 group exhibited a significantly higher mutation frequency for KRAS (84% vs. 57%) and CDKN2A (33% vs. 10%) compared to the G1 group. Targeting the KRAS/AMPK signalling axis may effectively weaken CSCs and enhance chemosensitivity to gemcitabine, thereby improving clinical outcomes in PDAC. In addition, we evaluated the mutation burden and intra‐tumour heterogeneity parameters including non‐silent mutation rate, aneuploidy score, fraction altered, and SNV neoantigens among the four groups, and we found that G4 group always had the highest values, while G1 group always had the lowest (Figure S4E). These results demonstrated that ARPC1B is significantly correlated with PDAC stemness, and the ARPC1B‐enriched high‐stemness subgroup exhibits a high frequency of driver gene mutations and increased intra‐tumour heterogeneity, which may explain the resistance to gemcitabine. Furthermore, a total of 9 PDAC datasets were merged (Figure S5A) and batch effect was removed (Figure S5B). A complex heatmap depicted the relationship between stemness, ARPC1B expression level, resistance to gemcitabine, and survival status across the 9 PDAC datasets (Figure S5C). The ARPC1B^+^ CSC cell population exhibited unique tumour function enrichment compared to other cell populations, such as mitotic spindle, epithelial‐mesenchymal transition, TGF‐β signalling, apical junction, and KRAS signalling up (Figure S5D). In silico ARPC1B knockout and GO enrichment analyses using the “scTenifoldKnk” tool in the specifically extracted C1 and C6 cell clusters showed various biological functions correlated with APRC1B in these CSC‐like cells (Figure S5E,F).

## 3 Targeting ARPC1B Can Sensitise Pancreatic Cancer to Gemcitabine Treatment

**FIGURE 2:**
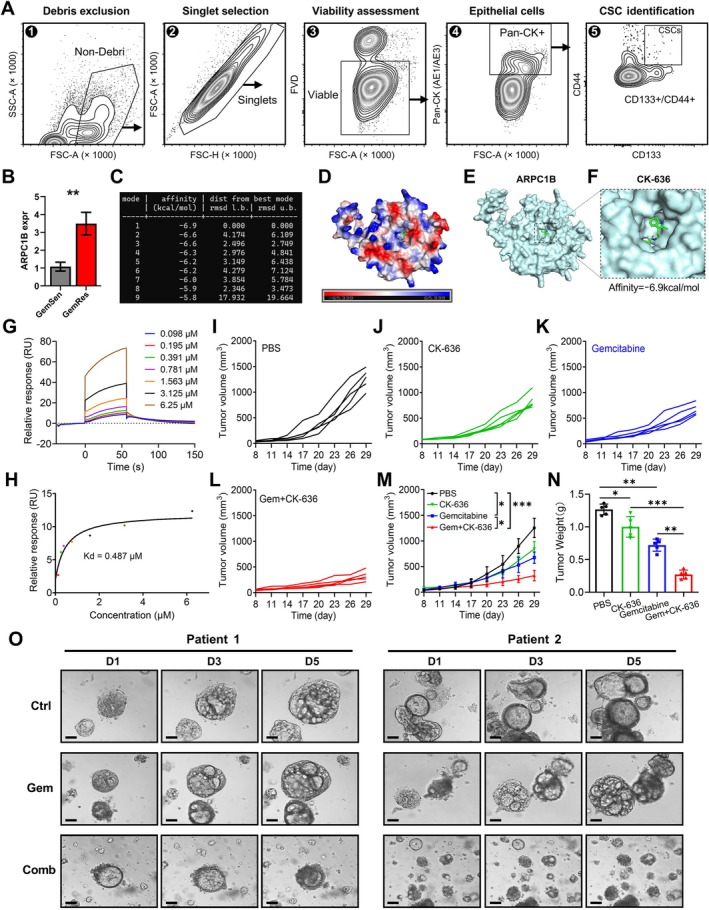
Targeting ARPC1B can sensitise pancreatic cancer to gemcitabine treatment. (A) Flow cytometry gating and sorting strategy for CSC identification and isolation, including debris exclusion, singlet selection, viability assessment, epithelial cell enrichment, and CSC identification. (B) ARPC1B expression was significantly upregulated in CSCs from gemcitabine‐resistant patients compared to sensitive ones. (C) Molecular docking results of CK‐636 and ARPC1B protein. Nine of calculations between CK‐636 and ARPC1B protein indicated the lowest binding energy of −6.9 kcal/mol. (D) Adaptive Poisson–Boltzmann Solver (APBS) analysis shows the overall charge of the ARPC1B protein surface and the positively charged active pocket where CK‐636 is located. (E, F) 3D structure of ARPC1B protein and binding details of CK‐636. (G) Surface plasmon resonance (SPR) analysis demonstrated a concentration‐dependent binding response between the analyte CK‐636 and the immobilised ligand ARPC1B, as evidenced by increasing response units (RUs) with higher analyte concentrations. The SPR graph exhibited rapid association and slow dissociation kinetics, indicative of a high‐affinity interaction between CK‐636 and ARPC1B. (H) The calculated equilibrium dissociation constant (Kd = 0.487 μM) further quantified the binding affinity. (I—L) In vivo antitumor evaluation by subcutaneous KPC‐Luc pancreatic tumour model of the control group, CK‐636‐treated group, gemcitabine‐treated group, and gemcitabine+CK‐636 combination‐treated group over a 4‐week period (*n* = 5). (M) Average tumour growth curves of different treatment groups. (N) Comparison of tumour weights among different groups. The combination‐treated group showed significantly smaller tumour weights compared to the other groups. (O) Tumour organoid growth was significantly inhibited in the combined therapy group compared to the control group and gemcitabine‐only group (scale bar: 100 μm). *p < 0.05; **p < 0.01; ***p < 0.001.

Pancreatic cancer tissues were obtained from the Pancreas Center of the First Affiliated Hospital of Nanjing Medical University and processed within two hours after biopsy. Detailed processing procedures of samples are provided in the supplementary file. Flow cytometry gating and sorting strategies were employed to identify and isolate CSCs in tumour tissues. As described in our previous studies [12, 13] and illustrated in Figure 2A, after the single‐cell suspension was prepared and stained with specific antibodies, the cells were subjected to flow cytometry sorting to isolate CSCs step‐by‐step: 1. Debris exclusion; 2. Singlet selection; 3. Viability assessment; 4. Epithelial cell enrichment (pan‐CK^+^); 5. CSCs identification. After obtaining CSCs from pancreatic cancer patients who are sensitive and resistant to gemcitabine, we performed RNA‐sequencing on these samples, and we found that ARPC1B expression was significantly increased in the CSCs from the gemcitabine‐resistant samples (*p* < 0.01, Figure 2B). We comprehensively researched the drug‐target database and found that CK‐636 could potentially target the ARPC1B protein. Molecular docking calculations were performed 9 times and revealed a minimum binding energy of −6.9 kcal/mol, indicating the tightest binding occurs at this energy level (Figure 2C). The Adaptive Poisson‐Boltzmann Solver (APBS) analysis was performed and showed that the ARPC1B protein's surface has an overall charge of 65.339, with the small molecule CK‐636 located in a positively charged active pocket (Figure 2D). The 3D structure of the ARPC1B protein and the detailed binding of CK‐636 to the site with the lowest affinity score were depicted in Figure 2E,F. Surface plasmon resonance (SPR) analysis demonstrated a concentration‐dependent binding response between the analyte CK‐636 and the immobilised ligand ARPC1B, as evidenced by increasing response units (RUs) with higher analyte concentrations (Figure 2G, Table S3). The SPR graph exhibited rapid association and slow dissociation kinetics, indicative of a high‐affinity interaction between CK‐636 and ARPC1B. Steady‐state analysis revealed a strong correlation between analyte concentration and binding signal, supporting the specificity of their interaction. The calculated equilibrium dissociation constant (Kd = 0.487 μM) further quantified the binding affinity (Figure 2H). Subsequently, we established gemcitabine‐resistant KPC‐Luc cells (denoted as GR‐KPC) for in vivo antitumor efficacy evaluation (Figure S6–S8). The GR‐KPC model was established by subcutaneously injecting 2 × 10^6^ GR‐KPC cells mixed with matrix gel (1:1 volume ratio) into 20 female C57BL/6 mice aged 6 to 8 weeks. Once the tumour size reached approximately 80 mm^3^, the tumour‐bearing mice were randomly divided into four groups and intravenously administered with PBS, CK‐636 alone, gemcitabine alone, or a combination of gemcitabine and CK‐636 (gemcitabine at 100 mg/kg, CK‐636 at 10 mg/kg), respectively, every other day. The body weight of each mouse in the four groups was measured and recorded as well (Figure S9). Figure 2I–L illustrate the changes in tumour volume of each mouse in the control group, CK‐636‐treated group, gemcitabine‐treated group, and the combination‐treated group over 4 weeks, and the mean values ± SD of the measured tumour volumes for each group were shown in Figure 2M. After 4 weeks, the mice were sacrificed, and the tumours were isolated and weighed. The results showed that the tumours in the combination‐treated group were significantly smaller than those in the PBS‐control group, CK‐636 alone, and gemcitabine alone groups (Figure 2N). Given that ARPC1B is enriched in a specific subgroup of CSCs, targeting the ARPC1B protein therefore means targeting this subpopulation of ARPC1B^+^ CSCs in pancreatic cancer. We then performed immunofluorescence staining for ARPC1B in treated vs. untreated subcutaneous tumours (Figure S10). The staining images demonstrated that Gem+CK‐636 combinations significantly reduced ARPC1B expression in tumour tissue. Gem+CK‐636 exhibited superior antitumor efficacy in the orthotopic pancreatic cancer model, further confirming the sensitising effect of CK‐636 on gemcitabine in drug‐resistant tumours (Figure S11). These findings demonstrated that targeting ARPC1B^+^ CSCs with CK‐636 can enhance the chemosensitivity to gemcitabine in pancreatic cancer.

Subsequently, we assessed the anti‐tumour efficacy of gemcitabine in combination with CK‐636 in PDAC organoids derived from patients resistant to the nab‐paclitaxel plus gemcitabine (AG) regimen. Resistance to AG was defined according to the following criteria: radiological assessments, including CT, MRI, or PET scans; a persistent elevation of CA199; the development of new distant metastases despite chemotherapy; and pathological evaluation of tumour poor differentiation. Following the protocols reported in previous studies [14], the patient‐derived organoids (PDOs) from two AG‐resistant PDAC patients were established and then treated with three different approaches: saline (control group), gemcitabine alone (30 nM), and a combination of gemcitabine (30 nM) and CK‐636 (50 nM). The saline group showed negligible impact on tumour organoid growth, while tumour organoid growth was significantly inhibited in the combined therapy group compared to the control group and gemcitabine‐only group (scale bar: 100 μm; Figure 2O). These findings suggested that the combination of gemcitabine and CK‐636 may enhance chemotherapeutic efficacy against AG‐resistant PDAC tumours.

## 4 Discussion

Pancreatic cancer patients often exhibit both intrinsic and acquired resistance to gemcitabine, which acts as a main therapeutic strategy for those advanced and metastatic cases [15]. In recent years, an increasing number of studies have indicated that CSCs are mainly responsible for this resistance [3]. CSCs are characterised by their self‐renewal capacity and multipotency, and play an important role in various malignant phenotypes such as tumour initiation, progression, and therapeutic resistance [4]. In pancreatic cancer, CSCs can be identified by specific surface markers such as CD133 and CD44. However, emerging evidence suggests that CSC populations are also heterogeneous [16]. Distinct subpopulations of CSCs exhibit distinct functional roles and treatment responses. In this study, we firstly focused on identifying a specific subpopulation of pancreatic CSCs that are particularly resistant to gemcitabine, and subsequently evaluated their potential as a novel biomarker and therapeutic target for pancreatic cancer.

Through a comprehensive analysis of scRNA‐seq data from PDAC samples, we identified the ARPC1B^+^ CSC subpopulation as specifically resistant to gemcitabine. ARPC1B was previously reported in the malignant progression of various cancer types. For example, Ke et al. reported that ARPC1B promotes ovarian cancer progression by regulating the AKT/PI3K/mTOR signalling pathway [17]. In addition, ARPC1B promotes radiotherapy resistance in glioblastoma [18], and high expression of ARPC1B indicates poor prognosis [19]. Moreover, Liu et al. reported that targeting ARPC1B can overcome immunotherapy resistance in glioblastoma by reversing pro‐tumorigenic macrophage polarisation [20]. In our study, we found ARPC1B is significantly upregulated in gemcitabine‐resistant CSCs compared to non‐resistant CSCs, and it is also significantly elevated in pancreatic cancer samples versus normal tissues. These findings suggest that ARPC1B^+^ CSCs are enriched in gemcitabine‐resistant cases and may serve as a potential biomarker of poor prognosis and a promising therapeutic target for gemcitabine‐resistant pancreatic cancer. Moreover, the correlation between ARPC1B expression and higher mutation burden and intra‐tumour heterogeneity further interprets its significance in chemoresistance.

We think that the identification of this specific CSC subpopulation and its association with gemcitabine resistance has significant clinical implications: targeting the ARPC1B^+^ CSC subpopulation may serve as an effective strategy to overcome the chemoresistance of pancreatic cancer cells to gemcitabine. In our in vivo and ex vivo experiments, the combination of gemcitabine and CK‐636, a compound identified through molecular docking analysis and SPR analysis as a potential ARPC1B‐targeting agent, demonstrated remarkable synergy in anti‐tumour efficacy. Subcutaneous and orthotopic pancreatic tumour models, and PDOs in the combination‐treated group were markedly smaller than those in the control or gemcitabine‐alone groups, which demonstrated that targeting ARPC1B^+^ CSCs by CK‐636 can sensitise pancreatic cancer to gemcitabine.

While existing research has highlighted the oncogenic role of ARPC1B in cancer progression, our findings offer new insights into its specific facet in gemcitabine resistance. ARPC1B's biological functions in CSCs and their interactions or communications with other cells within the tumour microenvironment need further investigation. We believe understanding these underlying mechanisms could facilitate the emergence of novel therapeutic strategies aimed at overcoming chemoresistance.

In conclusion, our study advances the understanding of the role of ARPC1B^+^ CSCs in pancreatic cancer chemoresistance. Targeting this specific CSC subpopulation shows the potential to enhance the efficacy of gemcitabine therapy. Future studies are wished to focus on further validation of ARPC1B as a robust therapeutic target through preclinical studies and eventually translating these findings into clinical trials. Additionally, exploring the combination of ARPC1B‐targeted therapies with other treatment modalities, such as immunotherapy or targeted therapy, may offer synergistic benefits in treating pancreatic cancer.

## Author Contributions

Y.W. and R.S. designed this study and wrote the first version of the manuscript. J.Z. and W.Z. conceived the idea, analysed the data, and drafted the figures. X.Z. collected and handled the samples. S.X. performed the animal experiment. X.W. performed the flow cytometry assay and analysis. Y.L., Z.Z., and C.Z. edited the manuscript. T.Z., W.S., and Z.Z. reviewed and made significant revisions to the manuscript. All authors read and approved the final manuscript.

## Ethics Statement

This work was approved by the Ethics Committee of the First Affiliated Hospital of Nanjing Medical University (2022‐SR‐624) and the Institutional Animal Care and Use Committee (IACUC) of Nanjing Medical University (IACUC‐2302020).

## Conflicts of Interest

The authors declare no conflicts of interest.

## Supporting information


**Figure S1:** The expression landscape of gene markers for 19 cell subtypes including immune cells such as natural killer T cells (NK/T), regulatory T cells (Tregs), CD8^+^ T cells, cycling T cells, CD4^+^ T cells, monocytes, neutrophils, macrophages, dendritic cells (DCs), and mast cells; epithelial cells such as pancreatic ductal cells (PDCs) and acinar cells (PACs); and stromal cells including pericytes, inflammatory cancer‐associated fibroblasts (iCAFs), and myofibroblasts (myCAFs).
**Figure S2:** The hdWGCNA algorithm was employed with a soft‐thresholding power (β = 8) at a scale‐free topology fit index of 0.90.
**Figure S3:** We observed that high ARPC1B expression and elevated stemness are significantly associated with worse survival, particularly among patients who are potentially gemcitabine‐resistant.
**Figure S4:** ARPC1B correlates with a higher mutation burden and increased intra‐tumour heterogeneity in pancreatic cancer. (A) Classification of 177 PAAD samples into four groups (G1‐G4) based on their stemness index and ARPC1B expression levels. (B) SsGSEA scores of gemcitabine resistance are significantly elevated in the Stemness^high^/ARPC1B^high^ group compared to the Stemness^low^/ARPC1B^low^ group. (C) Enrichment network illustrates the most significant biological processes associated with Stemness^high^/ARPC1B^high^. (D) Landscape plot of ARPC1B expression, stemness scores, somatic mutations, and clinicopathological characteristics. (E) Boxplots show the distributions of mutation burden and intra‐tumour heterogeneity parameters including non‐silent mutation rate, aneuploidy, fraction altered, and SNV neoantigen across the four groups. * *p* < 0.05; ** *p* < 0.01; *** *p* < 0.001.
**Figure S5:** (A) A total of 9 PDAC datasets were used. (B) Batch effect was removed during data integration. (C) A complex heatmap was generated to depict the relationship between stemness, ARPC1B expression level, resistance to gemcitabine, and survival status across the 9 PDAC datasets. (D) The ARPC1B^+^ CSC population exhibited unique tumour function enrichment compared to other cell populations, such as mitotic spindle, epithelial‐mesenchymal transition, TGF‐β signalling, apical junction, and KRAS signalling up. (E & F) In silico ARPC1B knockout and GO enrichment analyses in the specifically extracted C1 and C6 cell clusters (CSC‐like cells).
**Figure S6:** (A) Cell viabilities of various concentrations of gemcitabine against wild KPC‐Luc cells and our established GR‐KPC cells (*n* = 3). (B) Mean IC50 values of gemcitabine against wild KPC‐Luc cells and established GR‐KPC cells were 66.79 and 3.50 μM, respectively (*n* = 3). ** *p* < 0.01; *** *p* < 0.001.
**Figure S7:** Relative mRNA expression of drug resistance‐associated genes (Rrm1, Cda, Ent1, Abcc1) in wild KPC‐Luc and established GR‐KPC cells by RT‐qPCR (*n* = 3). * *p* < 0.05; ** *p* < 0.01.
**Figure S8:** (A) Cell viability curves of CK‐636 against GR‐KPC cells at different concentrations, the IC50 value of gemcitabine against GR‐KPC cells is 9.14 μM. (B) Cell viability of CK‐636 and Gem+CK‐636 combinations against GR‐KPC cells among different concentrations (X = gemcitabine in Gem+CK‐636 group). (C) The IC50 values of gemcitabine, CK‐636, and Gem+CK‐636 combinations against GR‐KPC cells were 3.50, 9.14, and 0.58 μM, respectively. In the Gem+CK‐636 combination, the mass ratio of gemcitabine and CK‐636 was 100:6. The cell viability data of gemcitabine were adopted from Figure S6. *n* = 3. * *p* < 0.05; *** *p* < 0.001.
**Figure S9:** The body weight of each mouse in the four groups (PBS‐treated control group, CK‐636‐only group, gemcitabine‐only group, and Gem+CK‐636 combination therapy group) was measured and recorded every other day (*n* = 5). ns: not significant.
**Figure S10:** (A) Immunofluorescence staining for ARPC1B (red) in treated vs. untreated subcutaneous tumours (scale bar: 50 μm). (B) ARPC1B (red) fluorescence‐based quantitative analysis from five individual figures. *** *p* < 0.001.
**Figure S11:** In vivo orthotopic pancreatic tumour model using GR‐KPC cells. (A) Bioluminescence images of different treated mice at day 8, day 18, and day 28 and (B) corresponding bioluminescence intensity quantification of tumour regions in different treatment groups. (C) Body weight changes of mice after different treatments every two days. *n* = 4. * *p* < 0.05; ** *p* < 0.01; ns: not significant.

**Table S1:** Gemcitabine resistance and stemness‐associated gene sets.

**Table S2:** Gemcitabine resistance associated hub genes.

**Table S3:** Kinetic affinity: ARPC1B and CK‐636.

## Data Availability

The data that support the findings of this study are available from the corresponding author upon reasonable request.
