# Supplementary material for: Targeting ARPC1B + Cancer Stem Cells to Sensitise Pancreatic Cancer to Gemcitabine Treatment
Source: Cell Prolif. 2025 Sep 3;58(12):e70125. doi: 10.1111/cpr.70125 (PMC12686117; doi:10.1111/cpr.70125)
Supplement: Supplementary file 1 — Figure S1: The expression landscape of gene markers for 19 cell subtypes including immune cells such as natural killer T cells (NK/T), regulatory T cells (Tregs), CD8+ T cells, cycling T cells, CD4+ T cells, monocytes, neutrophils, macrophages, dendritic cells (DCs), and mast cells; epithelial cells such as pancreatic ductal cells (PDCs) and acinar cells (PACs); and stromal cells including pericytes, inflammatory cancer‐associated fibroblasts (iCAFs), and myofibroblasts (myCAFs). Figure S2: The hdWGCNA algorithm was employed with a soft‐thresholding power (β = 8) at a scale‐free topology fit index of 0.90. Figure S3: We observed that high ARPC1B expression and elevated stemness are significantly associated with worse survival, particularly among patients who are potentially gemcitabine‐resistant. Figure S4: ARPC1B correlates with a higher mutation burden and increased intra‐tumour heterogeneity in pancreatic cancer. (A) Classification of 177 PAAD samples into four groups (G1‐G4) based on their stemness index and ARPC1B expression levels. (B) SsGSEA scores of gemcitabine resistance are significantly elevated in the Stemnesshigh/ARPC1Bhigh group compared to the Stemnesslow/ARPC1Blow group. (C) Enrichment network illustrates the most significant biological processes associated with Stemnesshigh/ARPC1Bhigh. (D) Landscape plot of ARPC1B expression, stemness scores, somatic mutations, and clinicopathological characteristics. (E) Boxplots show the distributions of mutation burden and intra‐tumour heterogeneity parameters including non‐silent mutation rate, aneuploidy, fraction altered, and SNV neoantigen across the four groups. * p < 0.05; ** p < 0.01; *** p < 0.001. Figure S5: (A) A total of 9 PDAC datasets were used. (B) Batch effect was removed during data integration. (C) A complex heatmap was generated to depict the relationship between stemness, ARPC1B expression level, resistance to gemcitabine, and survival status across the 9 PDAC datasets. (D) The ARPC1B+ CSC pop [file CPR-58-e70125-s002.docx]

**Supplementary file**

**Targeting ARPC1B^+^ Cancer Stem Cells to Sensitize Pancreatic Cancer to Gemcitabine Treatment**

Yang Wu^1^**^#^**, Jianpeng Zhang^2^**^#^**, Weixiong Zhu^3^**^#^**, Xinrui Zhu^4^**^#^**, Yi Liu^5^, Xin Wang^6^, Tianyu Zhao^7^, Chun Zhang^8^, Zili Zhang^9^, Wenjie Shi^10*^, Run Shi^11*^, Zhaokai Zhou^12*^, Shaohui Xu^13*^

1. Pancreas Center, The First Affiliated Hospital of Nanjing Medical University, Nanjing, China.
2. Department of Urology, The First Affiliated Hospital of Guangzhou Medical University, Guangzhou, China.
3. The Second Clinical Medical College, Lanzhou University, Lanzhou, China.
4. Department of Hepatobiliary and Pancreatic Surgery, The Third Affiliated Hospital of Soochow University, Changzhou, Jiangsu, China.
5. Department of Oncology, The Second Hospital of Dalian Medical University, Dalian, China.
6. Department of Oncology, The Affiliated Cancer Hospital of Nanjing Medical University, Jiangsu Cancer Hospital, Jiangsu Institute of Cancer Research, Nanjing, China.
7. Institute of Social Medicine and Epidemiology, Medical University of Graz, Graz, Austria.
8. Department of Gastroenterology, Affiliated Hospital of Nanjing University of Chinese Medicine, Nanjing, China.
9. State Key Laboratory on Technologies for Chinese Medicine Pharmaceutical Process Control and Intelligent Manufacture, Nanjing University of Chinese Medicine, Nanjing, China.
10. Molecular and Experimental Surgery, Clinic for General-, Visceral-, Vascular- and Transplantation Surgery, Medical Faculty and University Hospital Magdeburg, Otto-von-Guericke University, Magdeburg, Germany.
11. Department of Oncology, The First Affiliated Hospital of Nanjing Medical University, Nanjing, China.
12. Department of Urology, The Second Xiangya Hospital of Central South University, Changsha, China.
13. Institute of Functional Nano & Soft Materials (FUNSOM), Soochow University, Suzhou, China.

**#** These authors contributed equally to this work.

***** Correspondence should be sent to:

Shaohui Xu, Institute of Functional Nano & Soft Materials (FUNSOM), Soochow University, Suzhou, China. Email: shaohui.xu18@gmail.com

Zhaokai Zhou, Department of Urology, The Second Xiangya Hospital of Central South University, Changsha, China. Email: [13526278113@163.com](mailto:13526278113@163.com)

Run Shi, Department of Oncology, The First Affiliated Hospital of Nanjing Medical University, Nanjing, China. Email: [shirun@njmu.edu.cn](mailto:shirun@njmu.edu.cn)

Wenjie Shi, Molecular and Experimental Surgery, Clinic for General-, Visceral-, Vascular- and Transplantation Surgery, Medical Faculty and University Hospital Magdeburg, Otto-von-Guericke University, Magdeburg, Germany. Email: wenjie.shi@med.ovgu.de

**Materials and methods**

**Single-cell RNA-sequencing (scRNA-seq) data processing**

Six single-cell RNA (scRNA) sequencing datasets containing 109 pieces of samples related to pancreatic ductal adenocarcinoma (PDAC) were downloaded from the GEO database. Data preprocessing, analysis, and visualization were performed using “Seurat” (v4.4.1) and “Omicverse” (v1.6.5). Each dataset underwent quality control, filtering out doublets (“scDblFinder” package) and background contamination (“decontX” package). The filtered gene expression matrix was normalized using the “NormalizeData” function. Principal component analysis (PCA) identified 3,000 highly variable genes (HVGs), and principal components were selected based on their variance contribution to reach 80% variance. Batch effects were removed using the “RunBBKNN” method (“bbknnR” package), and cell clustering was performed at resolutions from 0.01 to 0.09 (step 0.01) and from 0.1 to 3 (step 0.1). Clustering results were annotated with classical markers and genes from the CellMarker 2.0 database [1].

The cell subcluster-specific markers were jointly identified using the “genesorteR” and “presto” packages. The gemcitabine sensitivity score for individual cells, “UP_UCell” was calculated using 80 upregulated genes linked to gemcitabine resistance via the “UCell” algorithm [2]. The stemness score for individual cells, “RRA_score”, was derived from 27 gene sets and calculated using the “UCell” algorithm followed by Robust Rank Aggregation algorithm (RRA). The gene sets are described in Table S1. The pseudotime analysis was performed using the “monocle2” package to reconstruct differentiation trajectories of cancer stem-like cells. For TCGA cohort, the corresponding stemness score were calculated using “mRNAsi” and obtained from the Cancer Stemness Online tool (<http://bio-bigdata.hrbmu.edu.cn/CancerStemnessOnline/>) [3].

**Bulk tissue data processing**

Normal pancreas transcriptome data were downloaded from the GTEx Portal (<https://www.gtexportal.org/home/>) [4]. The standardized transcriptome RNA-seq data, somatic mutation profiles, and clinical annotation files for pancreatic cancer were obtained from The Cancer Genome Atlas (TCGA) via the cBioPortal (https://www.cbioportal.org/). The R packages “maftools” and “ComplexHeatmap” were utilized for summarizing, analyzing, and visualizing the mutation profiles. Overall survival (OS) analysis of 852 PDAC patients from a multicenter-derived meta cohort was conducted by the Kaplan-Meier Plotter platform (<https://kmplot.com/analysis/>) [5].

**Sample processing and cell preparation**

Pancreatic cancer tissues were obtained from the Pancreas Center of the First Affiliated Hospital of Nanjing Medical University, and processed within two hours after biopsy. Tissues were minced and subjected to enzymatic digestion using a buffer containing collagenase and DNase at 37°C for 30-50 minutes. The cell suspension was dissociated by repeated pipetting and filtered through a 70-μm cell strainer to obtain a single-cell suspension. Subsequently, the single-cell suspension was washed twice with sterile phosphate-buffered saline (PBS) to remove residual enzymatic digestion buffer and debris. The cells were then resuspended in an appropriate volume of culture medium composed of RPMI 1640 supplemented with 10% fetal bovine serum (FBS) and 1% penicillin-streptomycin solution. The cells were counted and viability was assessed using trypan blue exclusion assay. Only cell suspensions with viability higher than 90% were used for downstream applications. When cryopreserving, cells were resuspended in freezing medium containing 90% FBS and 10% dimethyl sulfoxide (DMSO) at a concentration of 1×10⁶ cells/ml, and the freezing process was carried out gradually in a controlled-rate freezer to ensure high cell recovery rate upon thawing.

**Flow cytometry analysis**

The single-cell suspension was first analyzed using a flow cytometer (FACSAria II, BD Biosciences). Debris was excluded based on forward scatter (FSC) and side scatter (SSC) properties. Specifically, events with low FSC and SSC signals were gated out to remove cellular debris and small particles, ensuring that only intact cells were included in subsequent analyses. To discriminate singlet cells from doublets or aggregated cells, a singlet selection was performed based on FSC-height versus FSC-area signals. A gate was set to include only events with similar FSC-height and FSC-area values, effectively excluding doublets or cell clumps that exhibited discrepancies between these two parameters. Then, cell viability was assessed using Fixable Viability Dye (FVD). The dye was added to the cell suspension at a final concentration of according to the recommended protocol. After incubation for 15 minutes at room temperature, the cells were washed with PBS to remove excess dye. Viable cells were identified by their characteristic fluorescence emission, typically in the UV or near-infrared spectrum, depending on the dye used. Dead cells were excluded from the analysis based on their fluorescence intensity. For epithelial cell enrichment, cells were surface-stained with a pan-cytokeratin (pan-CK) antibody conjugated to a fluorescent marker, diluted in staining buffer (PBS containing 2% fetal bovine serum and 0.1% sodium azide). The cells were incubated with the antibody for 20 minutes at 4°C in the dark. After staining, the cells were washed twice with staining buffer to remove unbound antibody. Epithelial cells were identified and gated based on their positive pan-CK fluorescence signal, typically detected in the APC channel. To identify cancer stem cells (CSCs), the cells were further stained with a combination of antibodies targeting CSC markers (CD133 and CD44). The cells were incubated with the antibody cocktail for 20 minutes at 4°C in the dark. Following staining, the cells were washed twice with staining buffer. CSCs were identified based on the expression profile of CD133^+^/CD44^+^ using fluorescence detectors specific to each marker. The detailed protocols can be found in our previously published study [6].

**List of antibodies used in the above experiments.**

| **Antibody** | **Clone** | **Vender** | **Application** |
| --- | --- | --- | --- |
| Pan-CK | AE1/AE3 | NAAT | Flow Cytometry |
| CD133 | EMK08 | Thermo Fisher | Flow Cytometry |
| CD44 | IM7 | Thermo Fisher | Flow Cytometry |

**Establishment of Gemcitabine-Resistant KPC Cells**

Gemcitabine-resistant KPC-Luc cells (denoted as GR-KPC) were established via a stepwise dose-escalation method. Briefly, parental KPC-Luc cells were seeded in complete DMEM medium supplemented with 10% FBS and allowed to reach 70-80% confluency. Cells were initially treated with 5 nM gemcitabine for one week. Upon recovery and adaptation, the drug concentration was gradually increased every 7 days to 10 nM, 20 nM, 50 nM, 100 nM, and finally 200 nM. The dose escalation was halted when cells showed stable proliferation under 200 nM gemcitabine pressure without significant cell death. During the induction period, the medium was refreshed every 2-3 days with freshly prepared drug-containing medium, and cells were passaged as needed to maintain exponential growth. The resistance phenotype was validated by cell viability assays (CCK-8), comparing IC50 values between parental and resistant cells. Additionally, expression levels of drug resistance-associated genes (e.g., Rrm1, Cda, Ent1, Abcc1) were examined via RT-qPCR. GR-KPC cells were maintained in medium containing 100 nM gemcitabine and cultured without gemcitabine for at least 7 days before downstream assays.

**List of primers used in RT-qPCR test for Rrm1, Cda, Ent1, Abcc1.**

| Primers | Cat No | Supplier |
| --- | --- | --- |
| Rrm1 | QM20474S | Beyotime Biotechnology |
| Cda | QM41026S | Beyotime Biotechnology |
| Ent1 | QM30490S | Beyotime Biotechnology |
| Abcc1 | QM16886S | Beyotime Biotechnology |

**In vivo therapeutic evaluation in the GR-KPC subcutaneous pancreatic tumor model:** The therapeutic efficacy was evaluated by GR-KPC subcutaneous tumor model. The model was established by subcutaneously injecting 2×10^6^ GR-KPC cells mixed with matrix gel (1:1 volume ratio) into 6–8-week-old female C57BL/6 mice. Once the tumor size reached approximately 80 mm^3^, the tumor-bearing mice were randomly divided into three groups and intravenously administered with PBS, CK-636 (MCE, Cat No HY-15892), gemcitabine (Beijing Solarbio, Cat No G8970), or a combination of gemcitabine and CK-636 (dose: gemcitabine at 100 mg/kg, CK-636 at 6 mg/kg), respectively, every other day. Tumor size and body weight were recorded every three days. Tumor volume was calculated using the formula V = 1/2 ab^2^, where “a” represents the long diameter and “b” represents the short diameter. On day 29, all mice were sacrificed, and their tumors were isolated, weighed, and photographed. And tumors of PBS-treated group and Gem+CK-636-treated group were fixed in 4% paraformaldehyde, embedded in Optimal Cutting Temperature compound (OCT), and sectioned at 6-8 μm thickness. Tissue sections were subjected to immunofluorescence staining using primary antibodies against ARPC1B (Cat No E-AB-19260, Elabscience), followed by incubation with Cy5-conjugated secondary antibodies and counterstaining with DAPI.

**Establishment and treatment of pancreatic cancer patient-derived organoids (PDOs)**

The fresh tumor tissues were carefully washed with PBS to eliminate contaminants, then diced into 2-3 mm^3^ fragments using sterile scissors. The fragments were digested in MasterAim™ Tissue Enzyme Solution I (AIMINGMED, 100-051), diluted in advanced DMEM/F12 medium (Thermo Fisher Scientific), at 37°C for 1 hour with intermittent agitation. After initial digestion, additional Digestion™ Tissue Enzyme Solution I was applied to enhance tissue dissociation. Collected cells were resuspended in 30 µL Matrigel (356231, Corning, NY, USA) at 600 cells per well in a 48-well plate. The plate was incubated at 37°C for 5 minutes to solidify the Matrigel, forming a matrix for organoid growth. Cells were cultured in alkaline or complete medium. Alkaline medium contained advanced DMEM/F12, 10 mM HEPES, 1× GlutaMAX-I, 100 µg/mL Primocin, and 1× penicillin/streptomycin. Complete medium additionally included 500 nM A83-01, 10 µM Y-27632, 1.56 M N-acetylcysteine, 10 mM nicotinamide, 10 ng/mL FGF10, 1× B27 supplement, 10 µM forskolin, 30% Wnt3a conditioned medium, 2% R-corresponding conditioned medium, and 4% Noggin conditioned medium. The medium was refreshed every 3 days to maintain optimal growth conditions. The detailed protocols can be found in the previous studies [7, 8]. PDOs were treated for 48 h with three different approaches: saline (control group), gemcitabine alone (30 nM), and a combination of gemcitabine (30 nM) and CK-636 (50 nM). Photographs were taken on days 1, 3, and 5.

**Molecular docking analysis**

Protein structure files (PDB format) were obtained in the Protein Data Bank (RCSB PDB; <https://www.rcsb.org/>). Compound structures in SDF format were acquired from the PubChem database (<https://pubchem.ncbi.nlm.nih.gov/>), and their lowest-energy conformations were generated using Chem3D Pro 20.0 (PerkinElmer, USA). The optimized structures were converted to MOL format and subsequently prepared as ligand PDBQT files using AutoDockTools 1.5.7 (Scripps Research, USA). The molecular docking simulation was performed with AutoDock Vina 1.2.3, with the docking grid parameters centered on the co-crystallized ligand coordinates. The binding affinity (kcal/mol) and hydrogen bonding interactions between ligands and the target protein were selected as primary evaluation metrics, where lower binding energy and more hydrogen bonds indicate higher binding stability. Post-docking analysis included 3D interaction visualization using PyMOL 2.5.2 (Schrödinger LLC) and 2D interaction diagrams generated by LigPlot+ v.2.2.9 (EMBL-EBI).

**Surface plasmon resonance (SPR) measurement of CK-636 binding to ARPC1B**

Instruments: Binding kinetics were measured using a surface plasmon resonance (SPR) instrument (Biacore 8K, Cytiva).

**Compounds used in SPR**

| **Name** | **Supplier** | **MW (Da)** | **Buffer** |
| --- | --- | --- | --- |
| ARPC1B | FineTest (P2102) | 41500.0 | PBS, Trehalose |
| CK-636 | MCE (Cat No: HY-15892) | 287.32 | DMSO |

**Materials and methods used in SPR**

| **Experimental testing methods and parameter design** | |
| --- | --- |
| Chips | CM5 (Biacore) |
| Analyte | CK-636 |
| Analyte buffer | Cyto-PBS + T-DMSO PBS>7.4 (0.05% Tween-20, DMSO) |
| Regeneration buffer | 10mM Glycine-HCl（pH=2.0） |
| Analyte concentration | (0.10-6) μM |
| Injection speed of analyte | 20 μL/min |
| Association time | 60 s |
| Dissociation time | 100 s |
| Injection speed of regeneration | 200 μL/min |
| Regeneration time | 30 s |
| Temperature | 22℃ |

**Buffer information used in SPR**

| **Name** | **Details** |
| --- | --- |
| Activation buffer | 0.4 M EDC + 0.1 M NHS |
| Blocking buffer | 1 M Ethanolamine hydrochloride |
| Immobilization buffer | 10 mM Sodium Acetate, pH4.5 |
| Ligand buffer | HEPES: 10 mM HEPES, 150 mM NaCl, 3 mM EDTA, 0.005% Tween 20, pH 7.4 |
| Analyte buffer | 1% DMSO PBST: 1×PBS, 1% DMSO, 0.005% Tween 20 |
| Regeneration buffer | 10 mM Glycine-HCl（pH=2.0） |

**Chip preparation:** A mixture of 400 mM EDC and 100 mM NHS was freshly prepared immediately prior to injection. CM5 sensor chips (Biacore) were activated for 420 s with the EDC/NHS mixture at a flow rate of 10 μL/min.

**Ligand immobilization:** ARPC1B was prepared at a concentration of 20 μg/mL in immobilization buffer and injected into the sample channel (Fc2) at a flow rate of 10 μL/min, typically resulting in immobilization levels of approximately 12,600 response units (RU). The reference channel (Fc1) was left unmodified. After ligand immobilization, the chip surface was blocked by injecting 1 M ethanolamine hydrochloride at a flow rate of 10 μL/min for 420 s.

**Analyte injection (multi-cycle kinetics):** CK-636 was diluted in analyte buffer to final concentrations ranging from 0.098 to 6.25 μM (seven concentrations). Each concentration was sequentially injected over both Fc1 and Fc2 at a flow rate of 20 μL/min, with an association phase of 100 s followed by a dissociation phase of 180 s. Analyte buffer without CK-636 was used as a reference blank. All association and dissociation steps were performed using the same analyte buffer. A total of eight injection cycles were performed in ascending order of analyte concentration. The chip surface was regenerated after each interaction cycle.

**In vivo therapeutic evaluation in the GR-KPC orthotopic pancreatic tumor model:** An orthotopic pancreatic cancer model was established to evaluate the therapeutic efficacy, using GR-KPC cells. Briefly, 2×10^6^ luciferase-expressing GR-KPC cells suspended in 50 μL of PBS and Matrigel (1:1) were surgically implanted into the pancreas of C57BL/6 mice under isoflurane anesthesia. Eight days post-implantation, tumor engraftment was confirmed via bioluminescence imaging, and mice were randomly divided into four groups (n=4 per group): PBS control, gemcitabine (100 mg/kg), and Gem+CK-636 combination treatment. The treatments were administered intravenously every other day. On day 8, 18 and 28, mice were anesthetized, and tumor progression was monitored by bioluminescence imaging (IVIS Lumina III, PerkinElmer). The mouse weight was recorded every two days. After 20 days of treatment, mice were sacrificed.

**Statistical analysis**

Student’s t-test or one-way analysis of variance (ANOVA) was used to analyze differences among data subject to normal distribution and variance homogeneity; otherwise, the Mann-Whitney U test or Kruskal-Wallis test was used. Statistical significance was defined as a two-sided p-value or FDR-adjusted q-value below 0.05. The analyses were conducted using GraphPad Prism and R version 4.3.2.

**Supplementary figures and legends**


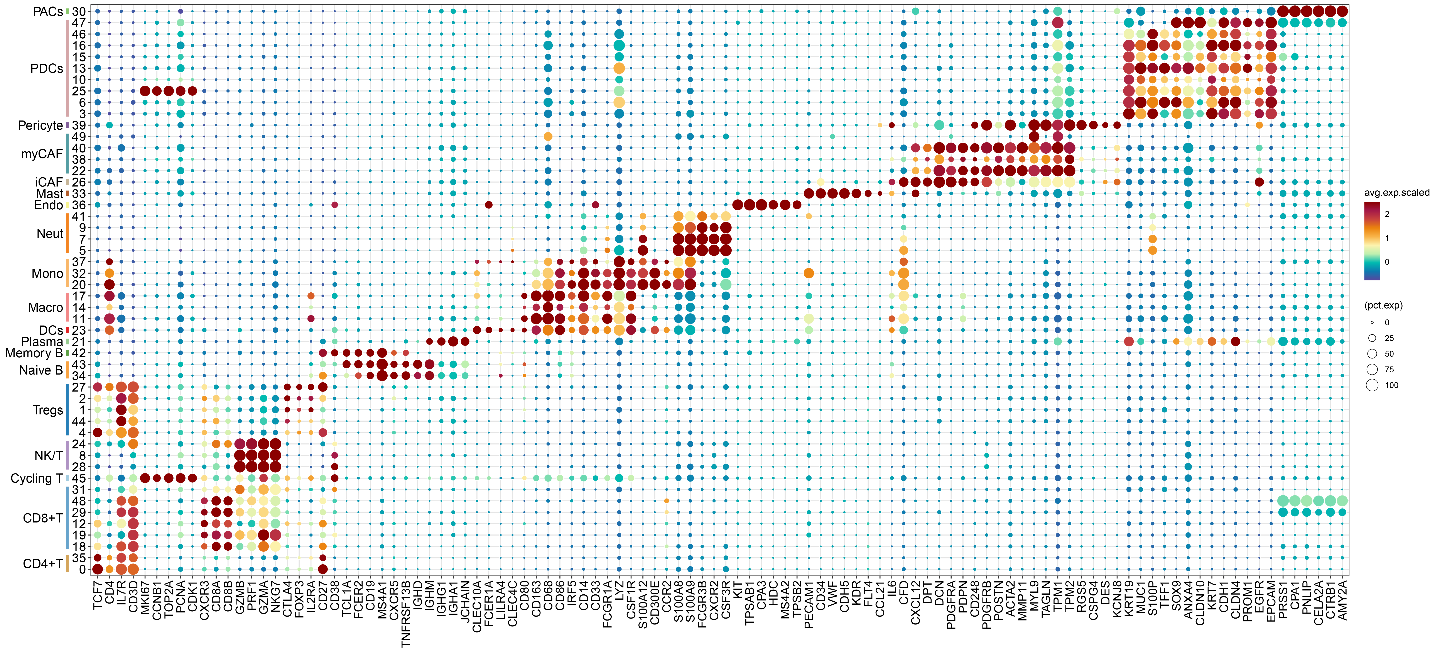


Figure S1: The expression landscape of gene markers for 19 cell subtypes including immune cells such as natural killer T cells (NK/T), regulatory T cells (Tregs), CD8^+^ T cells, cycling T cells, CD4^+^ T cells, monocytes, neutrophils, macrophages, dendritic cells (DC), and mast cells; epithelial cells such as pancreatic ductal cells (PDCs) and acinar cells (PACs); and stromal cells including pericytes, inflammatory cancer-associated fibroblasts (iCAFs), and myofibroblasts (myCAF).


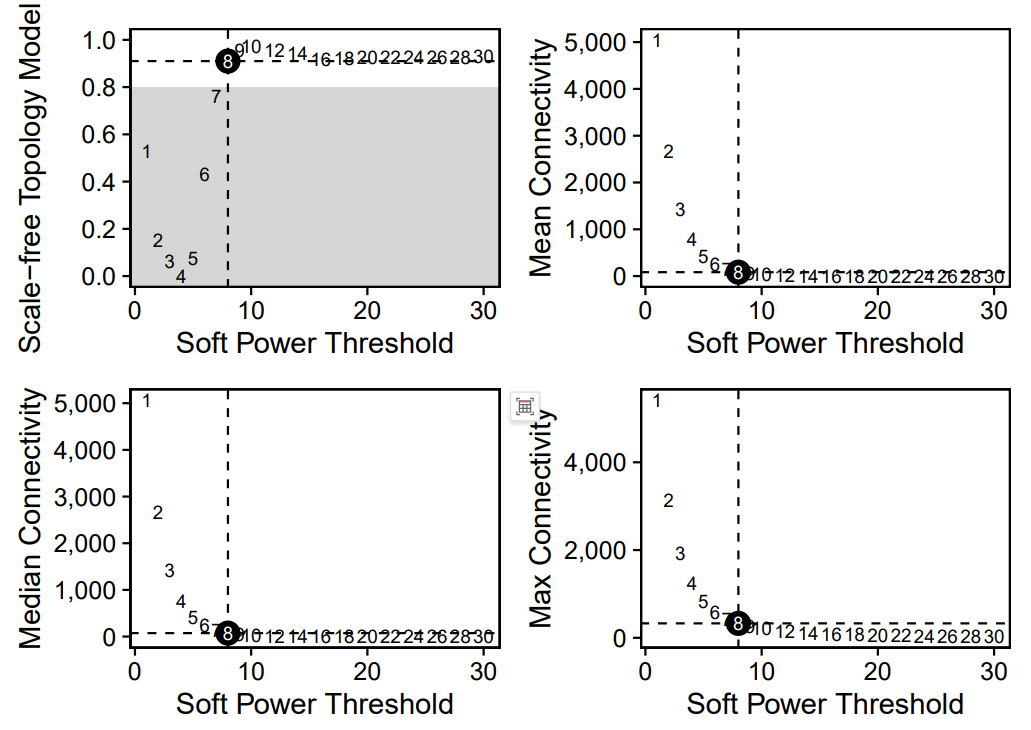


Figure S2: The hdWGCNA algorithm was employed with a soft-thresholding power (β = 8) at a scale-free topology fit index of 0.90.


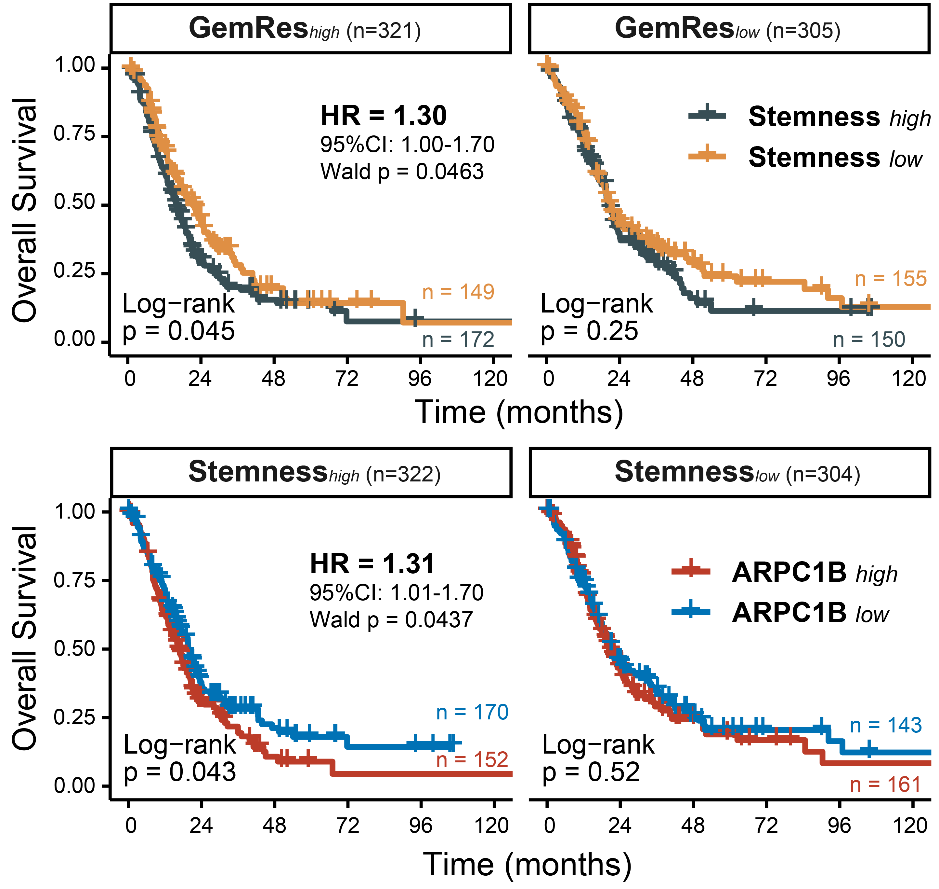


Figure S3: We observed that high ARPC1B expression and elevated stemness are significantly associated with worse survival, particularly among patients who are potentially gemcitabine-resistant.


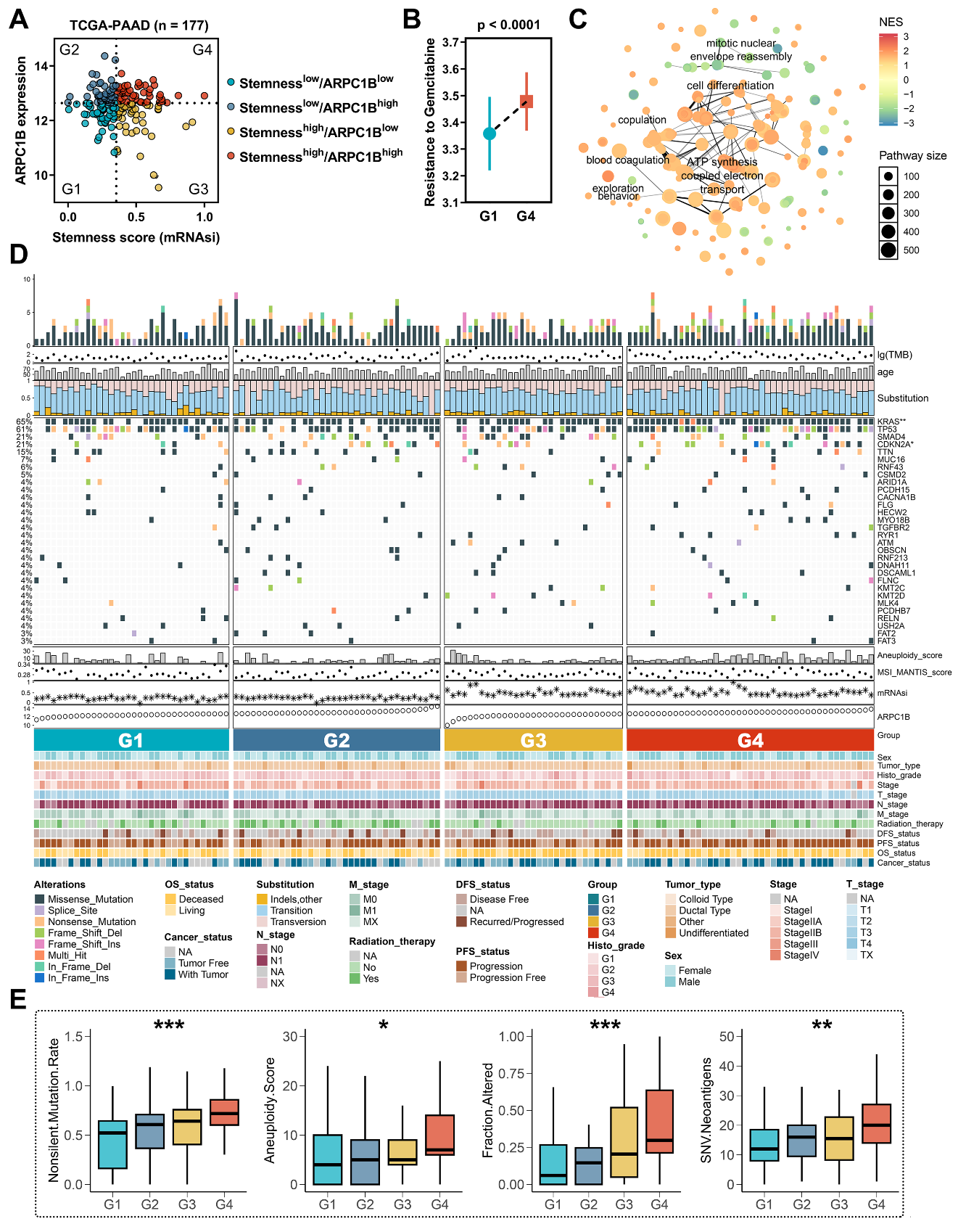


Figure S4: ARPC1B correlates with a higher mutation burden and increased intra-tumor heterogeneity in pancreatic cancer. (A) Classification of 177 PAAD samples into four groups (G1-G4) based on their stemness index and ARPC1B expression levels. (B) SsGSEA scores of gemcitabine resistance is significantly elevated in the Stemness^high^/ARPC1B^high^ group compared to the Stemness^low^/ARPC1B^low^ group. (C) Enrichment network illustrated the most significant biological processes associated with Stemness^high^/ARPC1B^high^. (D) Landscape plot of ARPC1B expression, stemness scores, somatic mutations, and clinicopathological characteristics. (E) Boxplots show the distributions of mutation burden and intra-tumor heterogeneity parameters including non-silent mutation rate, aneuploidy, fraction altered, and SNV neoantigen across the four groups. * p < 0.05; ** p < 0.01; *** p < 0.001.


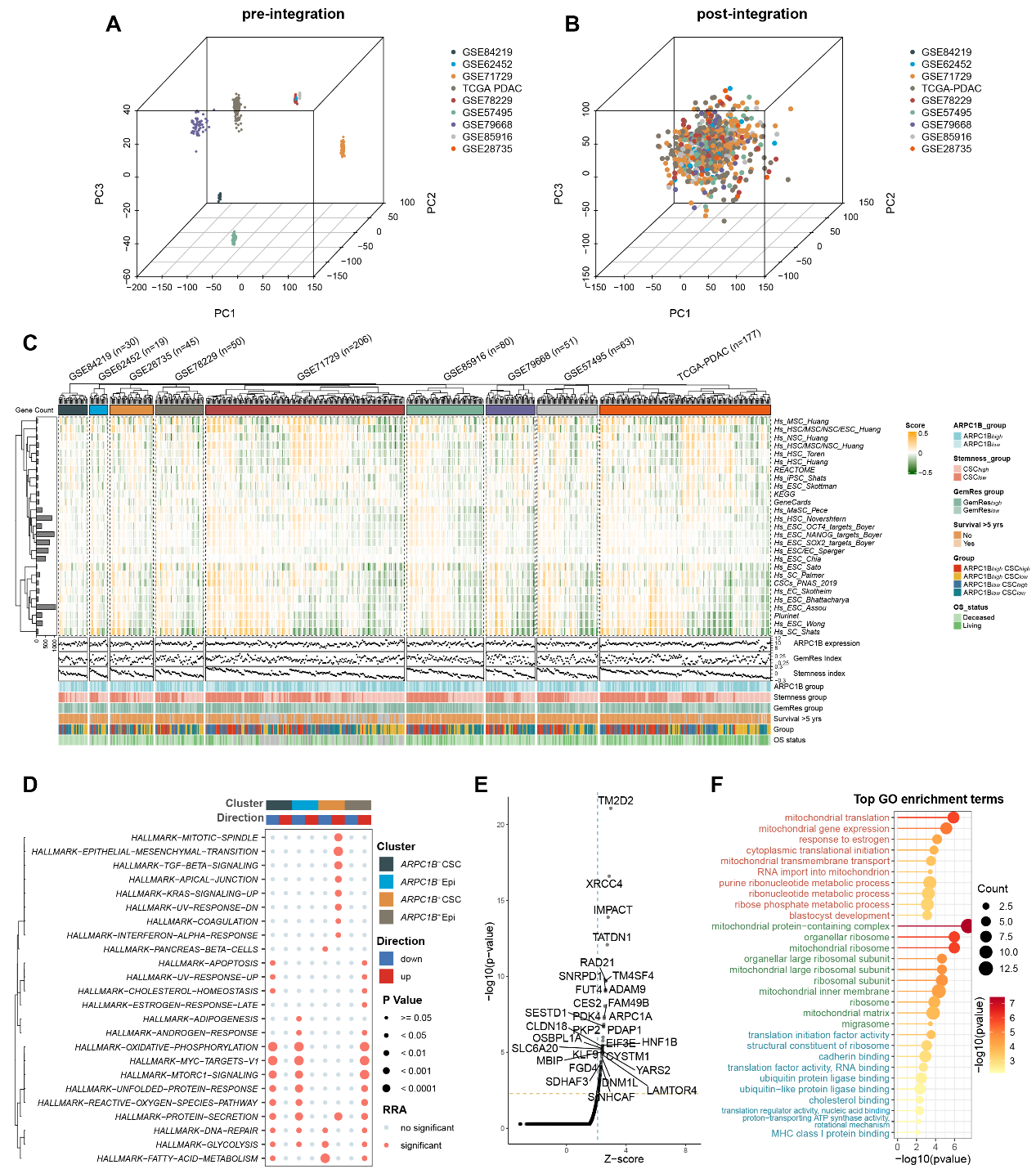


Figure S5: (A) A total of 9 PDAC datasets were used. (B) Batch effect was removed during data integration. (C) A complex heatmap was generated to depict the relationship between stemness, ARPC1B expression level, resistance to gemcitabine, and survival status across the 9 PDAC datasets. (D) The ARPC1B^+^ CSC population exhibited unique tumor function enrichment compared to other cell populations, such as mitotic spindle, epithelial-mesenchymal transition, TGF-β signaling, apical junction, and KRAS signaling up. (E & F) In silico ARPC1B knockout and GO enrichment analyses in the specifically extracted C1 and C6 cell clusters (CSC-like cells).


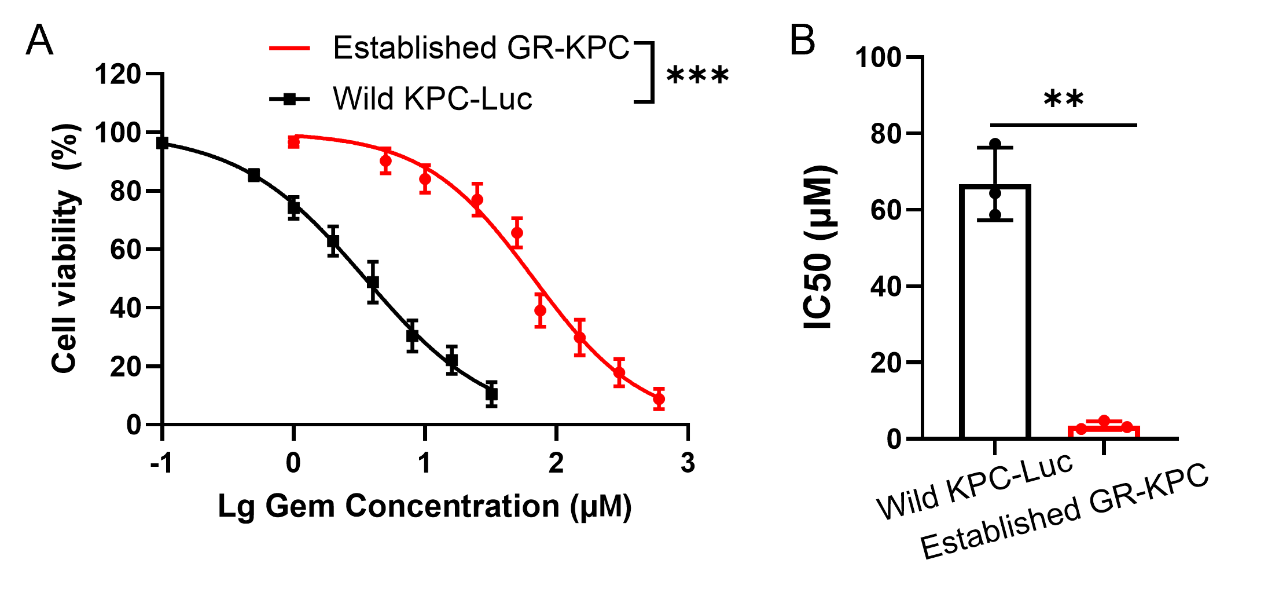


Figure S6: (A) Cell viabilities of various concentrations of gemcitabine against wild KPC-Luc cells and our established GR-KPC cells (n=3). (B) Mean IC50 values of gemcitabine against wild KPC-Luc cells and established GR-KPC cells were 66.79 and 3.50 μM, respectively (n=3). ** p < 0.01; *** p < 0.001.


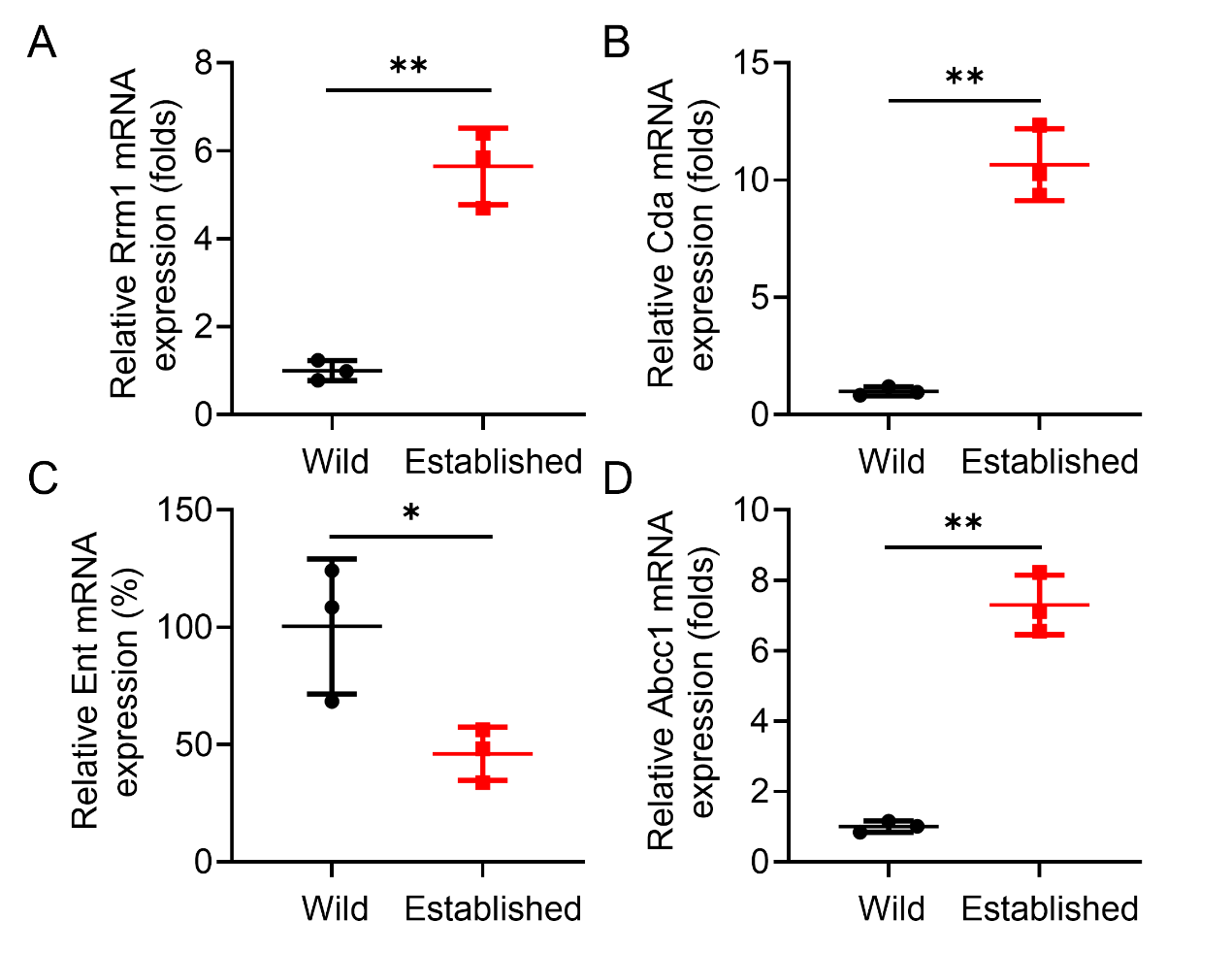


Figure S7: Relative mRNA expression of drug resistance-associated genes (Rrm1, Cda, Ent1, Abcc1) in wild KPC-Luc and established GR-KPC cells by RT-qPCR (n=3). * p < 0.05; ** p < 0.01.


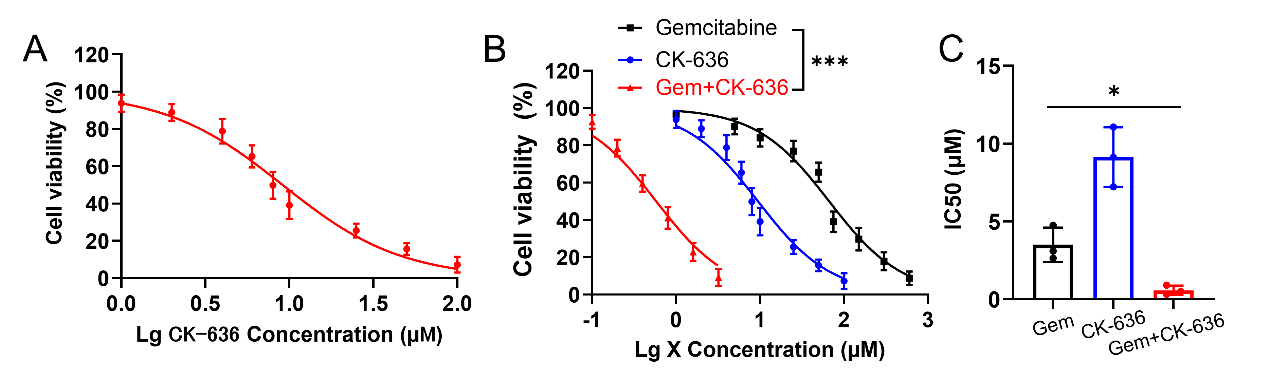


Figure S8: (A) Cell viability curves of CK-636 against GR-KPC cells at different concentrations, the IC50 value of gemcitabine against GR-KPC cells is 9.14 μM. (B) Cell viability of CK-636 and Gem+CK-636 combinations against GR-KPC cells among different concentrations (X=gemcitabine in Gem+CK-636 group). (C) The IC50 values of gemcitabine, CK-636 and Gem+CK-636 combinations against GR-KPC cells were 3.50, 9.14 and 0.58 μM, respectively. In the Gem+CK-636 combination, the mass ratio of gemcitabine and CK-636 was 100:6. The cell viability data of gemcitabine were adopted from Figure S6. n=3. * p < 0.05; *** p < 0.001.


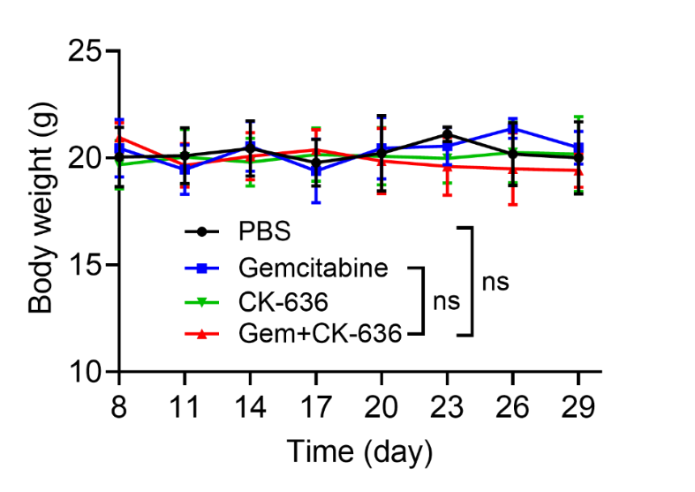


Figure S9: The body weight of each mouse in the four groups (PBS-treated control group, CK-636-only group, gemcitabine-only group, and Gem+CK-636 combination therapy group) was measured and recorded every other day (n=5). ns: not significant.


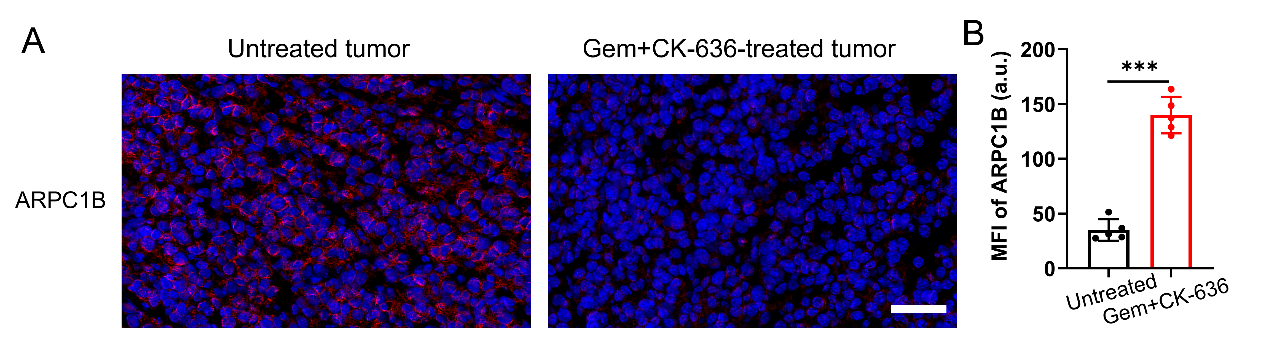


Figure S10. (A) Immunofluorescence staining for ARPC1B (red) in treated vs. untreated subcutaneous tumors (scale bar: 50 μm). (B) ARPC1B (red) fluorescence-based quantitative analysis from five individual figures. *** p < 0.001.


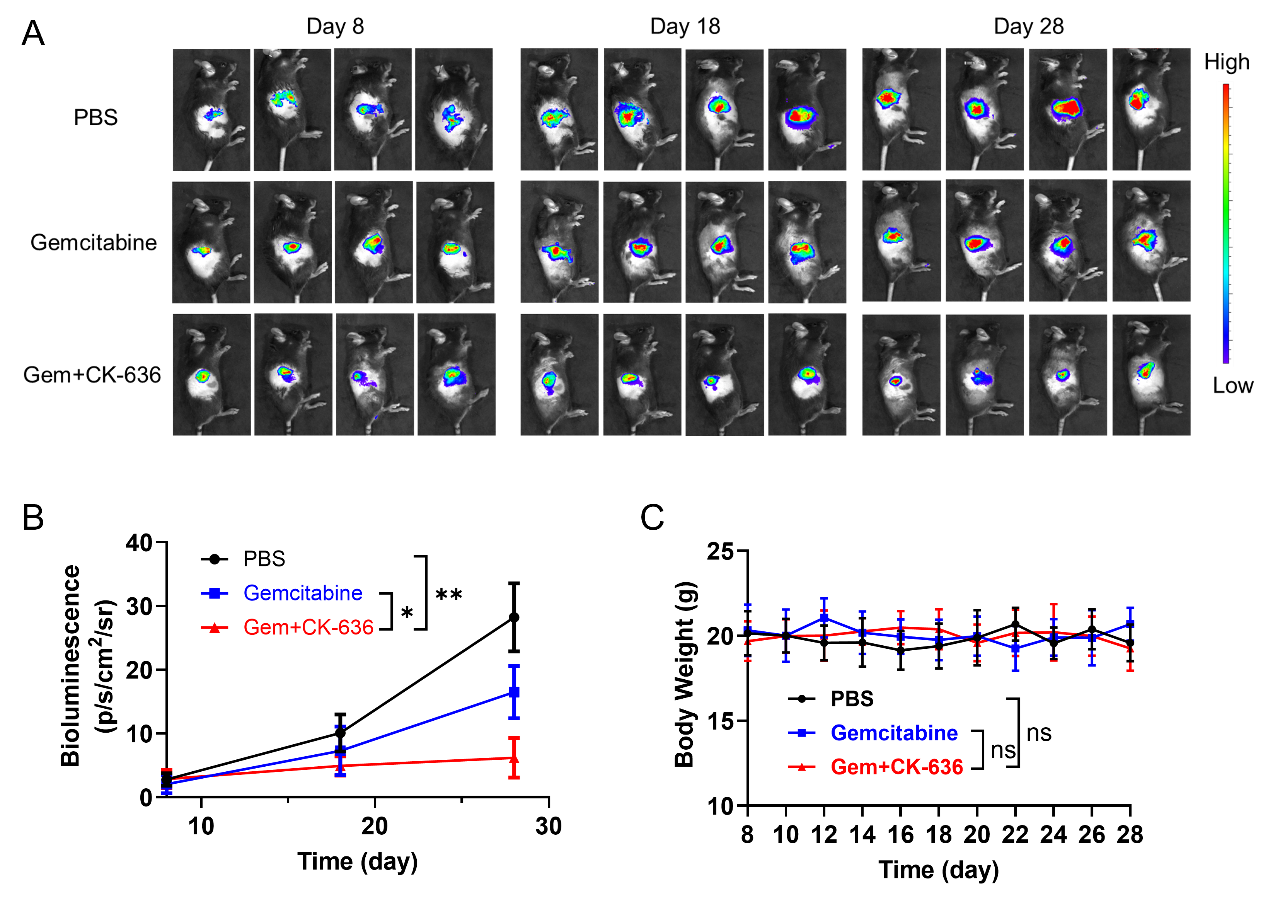


Figure S11: In vivo orthotopic pancreatic tumor model using GR-KPC cells. (A) Bioluminescence images of different treated mice at day 8, day18 and day 28 and (B) corresponding bioluminescence intensity quantification of tumor regions in different treatment groups. (C) Body weight changes of mice after different treatments every two days. n=4. * p < 0.05; ** p < 0.01; ns: not significant.

**References:**

1. Hu C, Li T, Xu Y, Zhang X, Li F, Bai J, Chen J, Jiang W, Yang K, Ou Q *et al*: **CellMarker 2.0: an updated database of manually curated cell markers in human/mouse and web tools based on scRNA-seq data**. *Nucleic Acids Res* 2023, **51**(D1):D870-D876.

2. Andreatta M, Carmona SJ: **UCell: Robust and scalable single-cell gene signature scoring**. *Comput Struct Biotechnol J* 2021, **19**:3796-3798.

3. Zhou W, Su M, Jiang T, Xie Y, Shi J, Ma Y, Xu K, Xu G, Li Y, Xu J: **Cancer Stemness Online: A Resource for Investigating Cancer Stemness and Associations with Immune Response**. *Genomics Proteomics Bioinformatics* 2024, **22**(4).

4. Consortium GT: **The Genotype-Tissue Expression (GTEx) project**. *Nat Genet* 2013, **45**(6):580-585.

5. Posta M, Gyorffy B: **Analysis of a large cohort of pancreatic cancer transcriptomic profiles to reveal the strongest prognostic factors**. *Clin Transl Sci* 2023, **16**(8):1479-1491.

6. Wang X, Wen S, Du X, Zhang Y, Yang X, Zou R, Feng B, Fu X, Jiang F, Zhou G *et al*: **SAA suppresses alpha-PD-1 induced anti-tumor immunity by driving T(H)2 polarization in lung adenocarcinoma**. *Cell Death Dis* 2023, **14**(11):718.

7. Boj SF, Hwang CI, Baker LA, Chio, II, Engle DD, Corbo V, Jager M, Ponz-Sarvise M, Tiriac H, Spector MS *et al*: **Organoid models of human and mouse ductal pancreatic cancer**. *Cell* 2015, **160**(1-2):324-338.

8. Xie CK, Liao CY, Lin HY, Wu YD, Lu FC, Huang XX, Wang ZW, Li G, Lin CF, Hu JF *et al*: **Sulindac (K-80003) with nab-paclitaxel and gemcitabine overcomes drug-resistant pancreatic cancer**. *Mol Cancer* 2024, **23**(1):215.
